# The chain mediation model of psychological resilience and social participation among stroke survivors with disability: the role of exercise adherence and physical disability

**DOI:** 10.1186/s40359-026-04242-w

**Published:** 2026-02-26

**Authors:** Xuan Zhou, Ying Wang, Lanshu Zhou

**Affiliations:** 1 School of nursing, Naval Medical University, 800 Xiangyin Road, Yangpu District, Shanghai, 200433 China; 2 Department of nursing, Shanghai First Rehabilitation Hospital, 349 Hangzhou Road, Yangpu District, Shanghai, 200090 China

**Keywords:** Stroke, Social participation, Psychological resilience, Exercise adherence, Participation satisfaction

## Abstract

**Objective:**

This study aimed to examine the chain mediating roles of exercise adherence and physical disability in the relationship between psychological resilience and social participation among stroke survivors with disability.

**Methods:**

A cross-sectional study was conducted with 271 stroke survivors recruited from five rehabilitation departments in Shanghai between March and June 2025. Participants were assessed with the Utrecht Scale for Evaluation of Rehabilitation-Participation (USER-P), 10-item Connor-Davidson Resilience Scale (CD-RISC-10), Exercise Adherence Questionnaire (EAQ), and modified Rankin Scale (mRS). Structural equation model (SEM) with bootstrap resampling was employed to test the hypothesized pathways, adjusting for potential confounders.

**Results:**

Psychological resilience significantly predicted all three dimensions of social participation. After adjusting for confounders, exercise adherence and physical disability jointly mediated the relationship for participation frequency (indirect effect = 0.050, 95% CI [0.018, 0.096], accounting for 13.7% of the total effect) and participation restrictions (indirect effect = 0.104, 95% CI [0.038, 0.197], explaining 9.97% of the total effect). For participation satisfaction, exercise adherence alone was a stronger mediator (indirect effect = 0.388, 95% CI [0.124, 0.685], representing 36.78% of the total effect) than the chain pathway including physical disability.

**Conclusion:**

Exercise adherence and physical disability mediate the association between psychological resilience and social participation in stroke survivors with disability, with adherence playing a particularly central role in enhancing participation satisfaction. Rehabilitation programs should incorporate resilience-building interventions and strategies to enhance exercise adherence to facilitate social participation.

## Introduction

Stroke is a major cause of long-term disability worldwide, especially in aging societies like China [1]. Many stroke survivors experience persistent motor impairments that significantly restrict their ability to perform daily activities and engage in social roles [2]. Social participation—defined as involvement in life situations—is increasingly acknowledged as a crucial objective in stroke rehabilitation, closely linked to quality of life and successful reintegration into the community [3]. However, social participation often remains limited among stroke survivors due to persistent physical deficits, psychological challenges, and environmental barriers [4, 5]. Reduced social participation is strongly associated with poorer quality of life, heightened loneliness, and an increased risk of depression [6, 7].

Physical disability is one of the most significant determinants of social participation in stroke patients [8]. Although stroke-related limb impairment is largely irreversible, rehabilitation exercises can, to some extent, help restore motor function and rebuild self-care capacity [9]. Therefore, adherence to rehabilitation exercise is widely considered a cornerstone for improving post-stroke physical disability and facilitating social participation. Nevertheless, poor adherence remains a common and serious challenge in clinical practice [10, 11]. Even when patients recognize the importance of exercise, many still struggle to maintain consistency due to various barriers. This “intention-behavior gap” considerably undermines the overall effectiveness of rehabilitation.

Recent studies have emphasized the role of psychological resilience in fostering adaptive recovery and improving functional outcomes after stroke [12, 13]. Psychological resilience is defined as the capacity to maintain or regain mental health and well-being in the face of adversity [14]. In chronic illness populations, higher resilience has been linked to better coping strategies, greater treatment adherence, and reduced anxiety and depression [15, 16]. Our previous cross-sectional study demonstrated that psychological resilience significantly predicts social participation after stroke [17]. However, the mechanisms through which resilience influences social participation remain poorly understood.

Evidence suggests that individuals with high psychological resilience exhibit enhanced stress-coping abilities—that is, they are more likely to deploy cognitive and behavioral strategies in challenging situations [18]. For stroke patients with disability, adherence to rehabilitation training represents a critical behavioral strategy for managing the impact of the disease. Thus, we hypothesize that exercise adherence and physical disability mediate the relationship between psychological resilience and social participation: resilience strengthens patients’ commitment to rehabilitation, which in turn improves physical disability and ultimately leads to increased social participation.

The purpose of this study is to investigate whether exercise adherence and physical disability mediate the relationship between psychological resilience and social participation in stroke survivors with disability. This research aims to provide evidence to inform targeted interventions designed to enhance social participation through resilience-building and multidisciplinary rehabilitation strategies.

## Methods

This study employed a cross-sectional design, adhered to the STROBE (Strengthening the Reporting of Observational Studies in Epidemiology) reporting guidelines, and was approved by the Ethics Committee of Shanghai First Rehabilitation Hospital (YK-2025-03-011). A cross-sectional design was adopted for preliminary mediation testing whether associations are consistent with theoretical predictions.

### Participants

From March to June 2025, a convenience sampling method was used to recruit stroke survivors with disability from five rehabilitation departments in Shanghai. The inclusion criteria were: (1) meeting the diagnostic criteria for stroke established by the Fourth National Cerebrovascular Disease Conference, confirmed by cranial CT or MRI, including both ischemic and hemorrhagic stroke [19]; (2) age ≥ 18 years; (3) stable vital signs and absence of other severe comorbidities such as cancer or organ failure; (4) disease duration ≥ 3 months; (5) presence of disability as indicated by a modified Rankin Scale (mRS) score between 1 and 4; (6) voluntary participation in the study. Exclusion criteria included: (1) language or communication barriers; (2) cognitive impairment (MoCA score < 26); (3) severe emotional disorders. The sample size was determined based on recommendations for mediation analysis [20]. Assuming a small to medium indirect effect size, a sample of 240 participants provides adequate power (> 0.80) to detect the mediated effect using bias-corrected bootstrapping. To account for an anticipated 10% invalid questionnaire response rate, the final target sample size was set at 240 / 0.90 ≈ 267 participants. The actual enrolled sample of 271 participants exceeded this requirement.

### Measurement

A self-designed general information questionnaire was employed to collect sociodemographic and disease-related information, including gender, age, education level, disease duration, stroke type, and stroke severity.

Social participation was measured using the Utrecht Scale for Evaluation of Rehabilitation-Participation (USER-P), developed by Dutch researchers [21]. The scale comprises three subscales: Participation Frequency, Participation Restrictions, and Participation Satisfaction, with a total of 32 items. The Frequency subscale is divided into two parts: Part 1 includes 4 items rated on a 6-point Likert scale (0 = never to 5 = ≥ 36 h per week), and Part 2 comprises 7 items also scored from 0 to 5 (0 = never to 5 = ≥ 19 times). The Restrictions subscale contains 11 items rated from 0 to 3 (0 = impossible to 3 = no difficulty), while the Satisfaction subscale includes 10 items rated from 0 to 4 (0 = very dissatisfied to 4 = very satisfied). The USER-P does not yield a total score; instead, raw scores for each subscale are converted to a normalized score ranging from 0 to 100 using specific algorithms. Higher scores indicate greater participation frequency, fewer restrictions, and higher satisfaction. The Frequency subscale reflects objective aspects of participation, whereas the Restrictions and Satisfaction subscales capture subjective experiences [22]. The Chinese version of the USER-P has been validated specifically in stroke rehabilitation populations [23]. Cronbach’s α for the subscales ranged from 0.727 to 0.969, and test-retest reliability coefficients ranged from 0.734 to 0.824. The content validity index (CVI) was between 0.954 and 0.983 [23].

Psychological resilience was assessed using the 10-item Connor-Davidson Resilience Scale (CD-RISC-10), originally developed by Connor and Davidson [24] and later simplified by Campbell-Sills and Stein. Responses are recorded on a 4-point Likert scale ranging from 1 (“not true at all”) to 4 (“true nearly all the time”). The total score ranges from 10 to 40, with higher scores indicating greater psychological resilience. The scale has shown good reliability and validity across diverse populations, with reported Cronbach’s α of 0.94, split-half reliability of 0.89, and item-total correlations between 0.74 and 0.81 [25, 26].

Exercise adherence was measured with the Exercise Adherence Questionnaire (EAQ), developed by Lin et al. in 2013 [27]. It includes 14 items across three dimensions: engagement in physical exercise, monitoring of exercise effects, and active seeking of exercise advice. Each item is rated on a 4-point Likert scale, ranging from 1 (“cannot do at all”) to 4 (“can do completely”), yielding a total score between 14 and 56. The EAQ has been validated in stroke survivors [28]. It demonstrates good psychometric properties, including content validity (0.95), structural validity, criterion validity, test-retest reliability (ranging from 0.778 to 0.850), and a Cronbach’s α of 0.923 [28].

Physical disability was evaluated using the mRS, initially designed by Rankin in 1957 and refined by Warlow in 1988 [29]. The mRS assesses functional independence on a 7-point ordinal scale ranging from 0 (no symptoms) to 6 (death). A score of 0 indicates no neurological symptoms; 1 indicates minor symptoms without significant functional impairment; 2 reflects slight disability that prevents some activities but allows self-care; 3 denotes moderate disability requiring assistance with certain tasks yet retaining the ability to walk independently; 4 corresponds to moderately severe disability characterized by the inability to walk without assistance and requiring help with daily activities; 5 represents severe disability where the patient is bedridden, incontinent, and requires constant nursing care; and 6 indicates death.

### Process

Trained researchers administered the questionnaires in either paper or electronic format. Prior to completion, participants were provided with instructions regarding the requirements and precautions. The researchers verified the questionnaires for completeness and provided assistance, when necessary, to help participants fill in any missing responses. For those with visual impairments, the questionnaires were verbally administered and responses were documented by the researchers.

### Data analysis

Statistical analyses were performed using SPSS 27.0 and Mplus 8.4. Categorical data are presented as numbers and percentages, while continuous data that did not follow a normal distribution are summarized as median and interquartile range. The Mann-Whitney U test and the Kruskal-Wallis H test were used to examine the associations between demographic characteristics and the frequency, restrictions, and satisfaction of social participation.

Structural equation model (SEM) was conducted in Mplus 8.4 to investigate the mediating effects of exercise adherence and physical disability on the relationship between psychological resilience and social participation. The model was estimated using maximum likelihood estimation, and bootstrap resampling with 5000 iterations was applied to obtain confidence intervals for indirect effects. Variables showing significant association (*p* < 0.05) in univariate analyses were included in the model to control for potential confounding effects on social participation outcomes.

Model fit was assessed using the following standards: χ²/df < 3.00 indicated acceptable fit; Comparative Fit Index (CFI) and Tucker–Lewis Index (TLI) values greater than 0.90 were considered acceptable, and above 0.95 indicated good fit [30]; Root Mean Square Error of Approximation (RMSEA) and Standardized Root Mean Square Residual (SRMR) values below 0.08 indicated acceptable fit, and below 0.05 indicated good fit [31]. A two-tailed p-value < 0.05 was considered statistically significant for all analyses.

## Results

### Basic characteristics of the study participants

The general characteristics of the participants are presented in Table 1. Among the 271 stroke survivors with disability, 171 (63.1%) were male and 100 (36.9%) were female. Regarding age distribution, 41 participants (15.1%) were under 60 years old, 164 (60.5%) were aged 60–74, and 66 (24.4%) were 75 years or older. In terms of educational attainment, the majority (76.4%) had completed secondary education, while 10.0% had primary education or less, and 13.7% had attained tertiary education or higher.

**Table 1:** Basic characteristics of participants (*N* = 271)

Variables	*N* (%)	Participation frequency			Participation restriction			Participation satisfaction		
		Median (Q1, Q3)	Test statistic	*p*	Median (Q1, Q3)	Test statistic	*p*	Median (Q1, Q3)	Test statistic	*p*
Age			7.006^†^	0.030		8.812^†^	0.012		4.184^†^	0.123
<60 years	41 (15.1)	9.10 (3.60, 16.40)			39.40 (18.20, 51.50)			45.00 (25.00, 57.50)		
60–74 years	164 (60.5)	3.60 (0.00, 12.70)			19.70 (6.10, 42.40)			30.00 (20.00, 50.00)		
≥ 75 years	66 (24.4)	4.55 (0.00, 10.90)			15.20 (0.00, 42.40)			30.00 (20.00, 50.00)		
Gender			8527.000^‡^	0.970		8430.500^‡^	0.847		7865.000^‡^	0.270
Male	171 (63.1)	5.50 (0.00, 12.70)			21.20 (6.10, 42.40)			30.00 (20.00, 50.00)		
Female	100 (36.9)	5.50 (0.00, 12.70)			21.20 (1.50, 45.50)			30.00 (18.75, 50.00)		
Educational background			19.322^†^	<0.001		25.008^†^	<0.001		10.521^†^	0.005
Primary school and below	27 (10.0)	0.00 (0.00, 5.50)			9.10 (0.00, 21.20)			30.00 (17.50, 38.75)		
Middle school	207 (76.4)	3.60 (0.00, 12.70)			21.20 (6.10, 42.40)			30.00 (20.00, 50.00)		
University and above	37 (13.7)	12.70 (5.50, 21.80)			42.40 (30.30, 60.60)			50.00 (30.00, 60.00)		
Spousal status			3558.500^‡^	0.370		3847.000^‡^	0.849		3814.000^‡^	0.788
Yes	238 (87.8)	5.50 (0.00, 12.70)			21.20 (6.10, 42.40)			30.00 (20.00, 50.00)		
No	33 (12.2)	5.50 (0.00, 14.50)			21.20 (6.10, 45.50)			40.00 (20.00, 47.50)		
Employed before disease onset			3898.500^‡^	0.002		4301.000^‡^	0.032		4658.500^‡^	0.158
Yes	48 (17.7)	9.10 (4.55, 15.45)			34.85 (13.65, 45.50)			37.50 (23.75, 52.50)		
No	223 (82.3)	3.60 (0.00, 12.70)			18.20 (6.10, 42.40)			30.00 (20.00, 50.00)		
Caregiver			0.430^†^	0.807		2.774^†^	0.250		1.531^†^	0.465
No caregiver	2 (0.7)	10.00 (0.00, 20.00)			33.35 (0.00, 66.70)			21.25 (0.00, 42.50)		
Care provided by relatives	140 (51.7)	5.50 (0.00, 12.70)			27.30 (6.10, 50.00)			31.25 (20.00, 57.50)		
Care provided by nursing assistant	129 (47.6)	5.50 (0.00, 12.70)			18.20 (6.10, 42.40)			30.00 (20.00, 50.00)		
Family financial situation			11.739^†^	0.003		9.773^†^	0.008		4.896^†^	0.086
Poor	16 (5.9)	0.00 (0.00, 0.00)			0.00 (0.00, 15.15)			18.75 (11.25, 40.00)		
Average	148 (54.6)	5.50 (0.00, 10.90)			21.20 (6.10, 42.40)			30.00 (20.00, 50.00)		
Good	107 (39.5)	7.30 (0.00, 18.20)			27.30 (6.10, 48.50)			35.00 (21.25, 57.50)		
Stroke type			6121.500^‡^	0.010		6659.000^‡^	0.113		6805.500^‡^	0.183
Ischemic	192 (70.8)	3.60 (0.00, 12.70)			21.20 (3.00, 42.40)			30.00 (20.00, 50.00)		
Hemorrhagic	79 (29.2)	7.30 (1.80, 15.45)			24.20 (9.10, 47.00)			32.50 (21.25, 57.50)		
Disease course			9.332^†^	0.009		9.433^†^	0.009		13.180^†^	0.001
≤ 6 months	133 (49.1)	7.30 (0.00, 14.50)			30.30 (9.10, 48.50)			40.00 (22.50, 57.50)		
>6months and ≤ 12months	75 (27.7)	0.00 (0.00, 10.90)			15.20 (0.00, 33.30)			25.00 (15.00, 37.50)		
>12 months	63 (23.2)	5.50 (0.00, 11.80)			18.20 (9.10, 37.90)			30.00 (21.25, 50.00)		

Note: †Kruskal-Wallis H test; ‡Mann-Whitney U test

### Scores of social participation and other variables

Table 2 summarizes the scores for social participation, psychological resilience, rehabilitation exercise adherence, and physical disability among the participants. The median scores were 5.50 (0.00, 12.70) for social participation frequency, 21.20 (6.10, 42.40) for social participation restrictions, and 30.00 (20.00, 50.00) for social participation satisfaction.

**Table 2:** Descriptive statistics for study variables (*N* = 271)

Variables	Median (Q1, Q3)	Min-Max
Participation frequency	5.50 (0.00,12.70)	0–71
Participation restriction	21.20 (6.10, 42.40)	0-100
Participation satisfaction	30.00 (20.00, 50.00)	0-100
Psychological resilience	21 (15, 29)	0–40
Exercise adherence	40 (31, 44)	14–56
Physical disability	4 (3,4)	1–4

### Univariate analysis of social participation

Univariate analysis of factors associated with social participation is presented in Table 1. Social participation frequency was associated with age, educational background, pre-stroke employment status, family financial situation, type of stroke, and disease course. Social participation restrictions were linked to age, educational level, pre-stroke employment, financial status, and disease course. Social participation satisfaction was associated with educational background and disease course.

### Pathway analysis of the effect of psychological resilience on social participation in stroke patients with physical disability

**Fig. 1:**
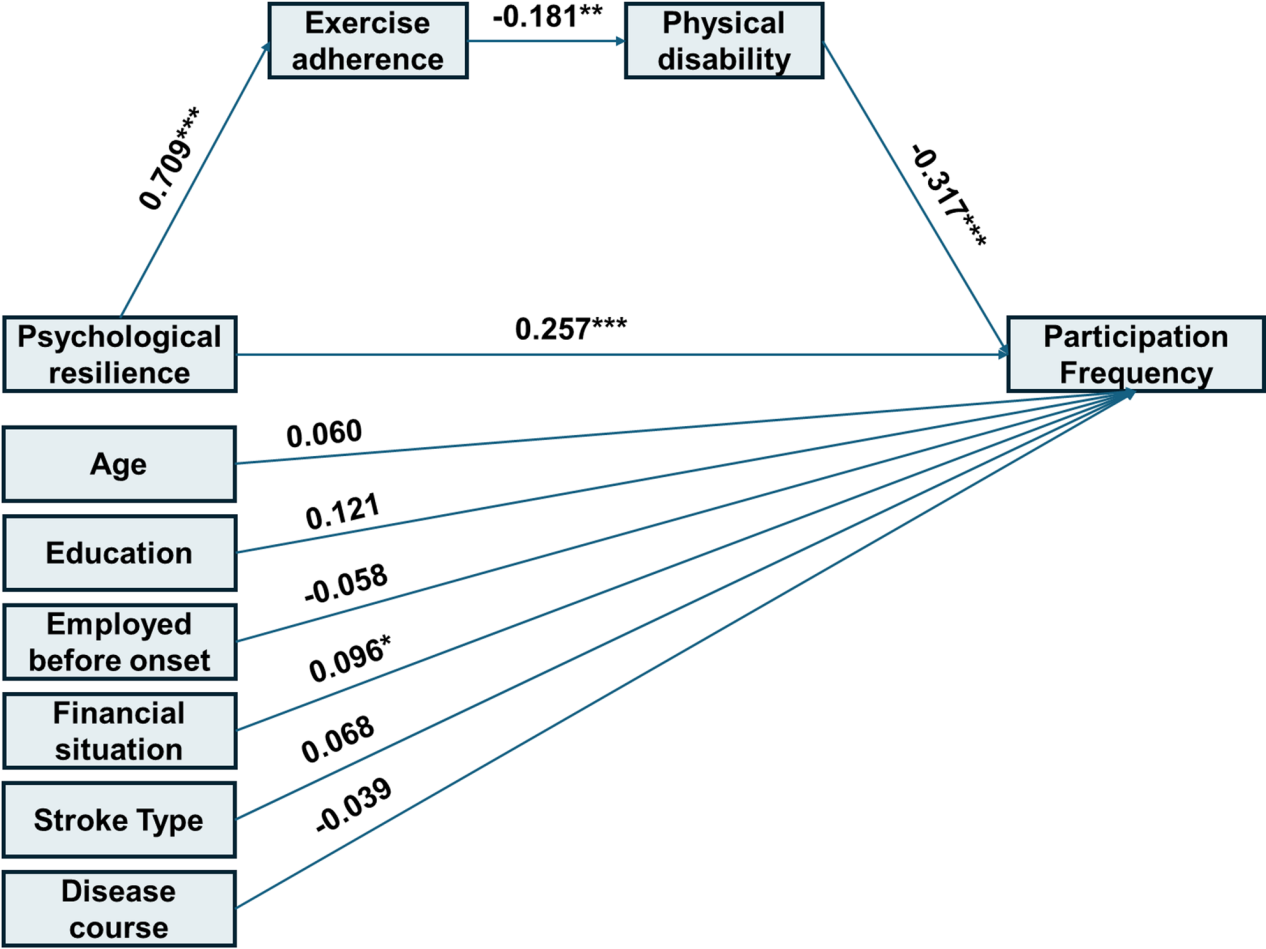
Standardized path coefficients for the serial mediation model predicting participation frequency

The SEM evaluating the influence of psychological resilience on social participation frequency is presented in Fig. 1. Although the chi-square test was significant, χ²(14) = 26.482, *p* = 0.022, the large sample size (*N* > 200) supports the use of alternative fit indices. These indicated a good model fit: χ²/df = 1.892, RMSEA = 0.057, SRMR = 0.051, CFI = 0.956, and TLI = 0.925. The chain mediation model accounted for 26.0% of the variance in social participation frequency (R² = 0.260). As detailed in Table 3, the total effect of psychological resilience on social participation frequency was 0.365 (95% CI: [0.234, 0.512]). The direct effect was 0.315, and the indirect effect through the pathway psychological resilience → exercise adherence → physical disability → social participation frequency was 0.050, representing 13.7% of the total effect.

**Table 3:** Parameter estimates for the serial mediation model predicting participation frequency (*N* = 271)

Paths	B	95%CI	β	*p*
Direct paths				
R → PF	0.315	[0.185, 0.455]	0.257	<0.001
EA → D	-0.021	[-0.033, -0.008]	-0.181	0.002
D → PF	-3.211	[-4.658, -1.988]	-0.317	<0.001
R → EA	0.742	[0.631, 0.834]	0.709	<0.001
Covariates on PF				
Age → PF	1.093	[-1.563, 3.666]	0.060	0.417
Work → PF	-1.671	[-6.281, 2.912]	-0.058	0.475
Education → PF	2.771	[-0.412, 5.928]	0.121	0.093
Family financial situation → PF	1.832	[0.116, 3.626]	0.096	0.043
Stroke type →PF	1.423	[-1.364, 4.740]	0.068	0.357
Disease course → PF	-0.031	[-1.346, 1.122]	-0.039	0.953
Indirect and total effect				
R → EA → D→ PF	0.050	[0.018, 0.096]	0.041	0.011
R →PF (total effect)	0.365	[0.234, 0.512]	0.298	<0.001

Note: *R *psychological resilience, *PF *participation frequency, *EA *exercise adherence, *D *physical disability, *B *unstandardized estimates, *β *standardized estimates

**Fig. 2:**
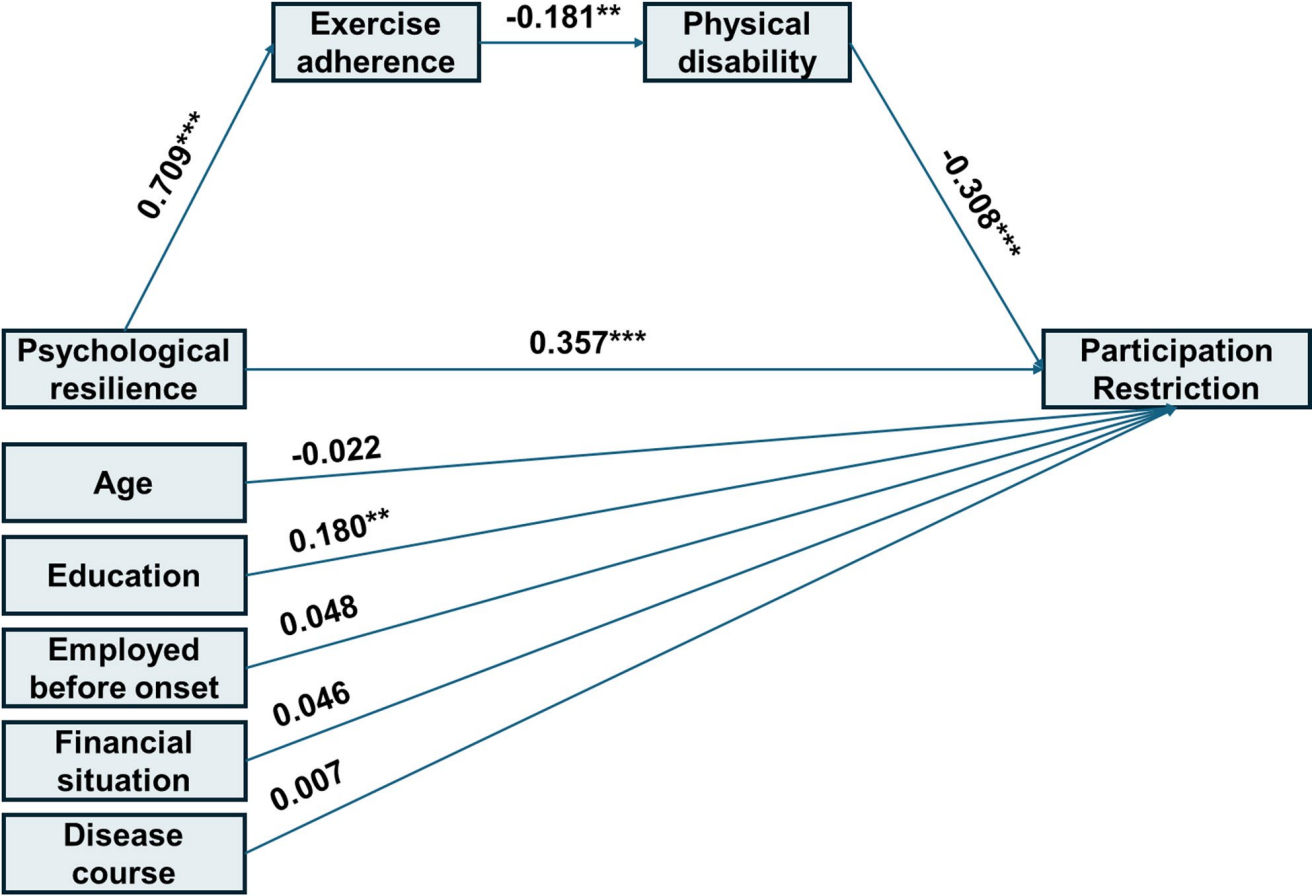
Standardized path coefficients for the serial mediation model predicting participation restriction

For social participation restrictions, the SEM is shown in Fig. 2. Despite a significant chi-square, χ²(12) = 29.520, *p* = 0.003, the fit indices suggested an acceptable model given the sample size: χ²/df = 2.460, RMSEA = 0.074, SRMR = 0.056, CFI = 0.945, and TLI = 0.904. This model explained 32.7% of the variance in social participation restrictions (R² = 0.327). The total effect of psychological resilience was 1.043 (95% CI: [0.788, 1.324]), with a direct effect of 0.939. The indirect effect through the chain mediation pathway (psychological resilience → exercise adherence → physical disability → social participation restrictions) was 0.104, accounting for 9.97% of the total effect (Table 4).

**Table 4:** Parameter estimates for the serial mediation model predicting participation restriction

Paths	B	95%CI	β	*p*
Direct paths				
R → PR	0.939	[0.677, 1.222]	0.357	< 0.001
R → EA	0.742	[0.631, 0.834]	0.709	< 0.001
EA → D	-0.021	[-0.033, -0.008]	-0.181	0.002
D → PR	-6.702	[-8.994, -4.597]	-0.308	< 0.001
Covariates on PR				
Age → PR	-0.854	[-5.879, 4.285]	-0.022	0.744
Work → PR	2.973	[-4.178, 10.632]	0.048	0.432
Education → PR	8.882	[2.613, 14.816]	0.180	0.004
Family financial situation → PR	1.877	[-1.856, 5.549]	0.046	0.319
Disease course → PR	0.012	[-3.847, 0.267]	0.007	0.995
Indirect and total effect				
R → EA→ D → PR	0.104	[0.038, 0.197]	0.040	0.010
R → PR (total effect)	1.043	[0.788, 1.324]	0.397	< 0.001

Note:* R* psychological resilience, *PR *participation restriction, *EA *exercise adherence, *D *physical disability, *B *unstandardized estimates, *β *standardized estimates

**Fig. 3:**
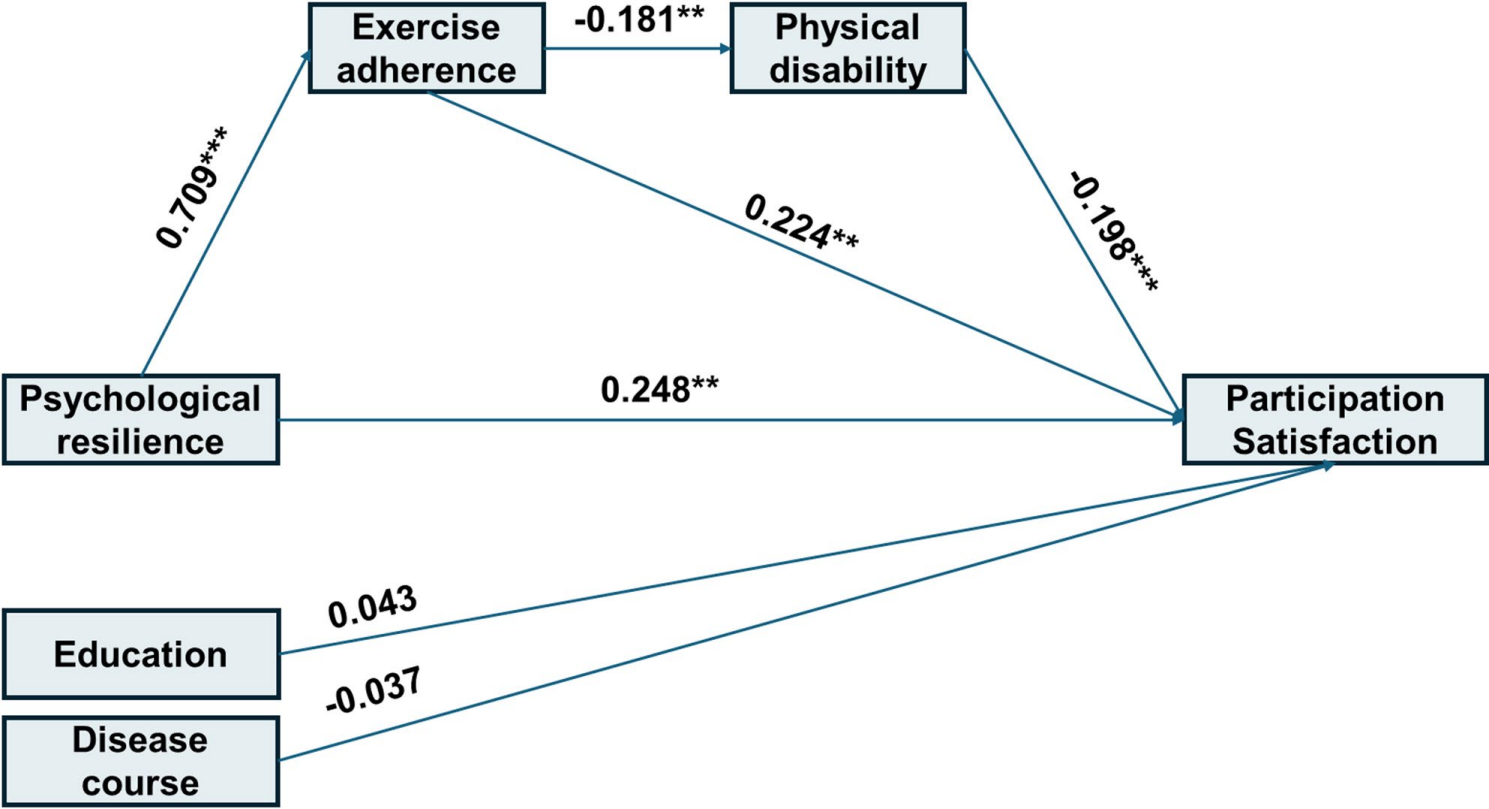
Standardized path coefficients for the mediation model predicting participation satisfaction

The model examining social participation satisfaction is depicted in Fig. 3. The chi-square was significant, χ²(5) = 14.307, *p* = 0.014, but other indices supported acceptable fit: χ²/df = 2.860, RMSEA = 0.083, SRMR = 0.045, CFI = 0.968, and TLI = 0.922. The model explained 26.9% of the variance (R² = 0.269). The total effect of psychological resilience was 1.055 (95% CI: [0.776, 1.333]). The direct effect was also 1.055. Two indirect pathways were identified: one through the chain psychological resilience → exercise adherence → physical disability → social participation satisfaction (effect = 0.062; 5.88% of total effect), and another through the simpler pathway psychological resilience → exercise adherence → social participation satisfaction (effect = 0.388; 36.78% of total effect). The overall mediation effect accounted for 42.65% of the total effect (Table 5).

**Table 5:** Parameter estimates for the serial mediation model predicting participation satisfaction

Paths	B	95%CI	β	*p*
Direct Paths				
R → PS	0.605	[0.200, 1.014]	0.248	0.004
R → EA	0.742	[0.631, 0.834]	0.709	< 0.001
EA → D	-0.021	[-0.033, -0.008]	-0.181	0.002
EA → PS	0.523	[0.172, 0.895]	0.224	0.006
D → PS	-3.993	[-6.252, -1.845]	-0.198	< 0.001
Covariates on PS				
Education → PS	1.952	[-3.223, 6.990]	0.043	0.459
Disease course → PS	-0.058	[-3.386, 0.803]	-0.037	0.968
Indirect and total effects				
R → EA → D → PS	0.062	[0.019, 0.135]	0.025	0.029
R → EA → PS	0.388	[0.124, 0.685]	0.159	0.007
R → PS (total effect)	1.055	[0.776, 1.333]	0.432	< 0.001

Note: *R *psychological resilience, *PS *participation satisfaction, *EA *exercise adherence, *D *physical disability, *B *unstandardized estimates,* β *standardized estimates

## Discussion

This study revealed that psychological resilience significantly and positively predicted all three dimensions of social participation—frequency, restrictions, and satisfaction—directly and through key mediating pathways among stroke survivors with physical disability. Exercise adherence and physical disability were identified as significant mediators in these relationships.

Specifically, exercise adherence and physical disability were found to play a chain mediating role in the relationship between psychological resilience and both the frequency and restrictions of social participation. Psychological resilience refers to an individual’s capacity for positive adaptation in the face of adversity [32]. Individuals with higher psychological resilience tend to adapt more effectively and rapidly to challenging circumstances. Basic research indicates that psychological resilience is not merely a psychological trait but a dynamic process involving neurobiological systems. For example, highly resilient individuals exhibit enhanced prefrontal cortex regulation [33, 34], which facilitates modulation of stress-induced negative emotions—such as fear and anxiety—and promotes flexible, goal-directed behavior rather than emotionally driven reactions. They also display a more efficient stress response system, capable of rapid activation under pressure and optimal cortisol release [35]. Moreover, neurobiological systems related to social bonding and emotional regulation—such as oxytocin and serotonin—function more effectively in highly resilient individuals [36, 37]. These mechanisms collectively support stronger stress-coping abilities, encompassing cognitive and behavioral strategies like problem-solving, seeking support, and finding meaning in adversity.

As is widely recognized, stroke is a sudden and highly disabling event. Previous qualitative studies have reported that some survivors describe it as “a bolt from the blue” [38]. Fortunately, motor impairments resulting from stroke can be partially restored through rehabilitation. Studies indicate that most stroke patients can regain the ability to perform self-care activities, despite residual disabilities [39]. When confronted with post-stroke physical disability, highly resilient individuals engage in rehabilitation exercises as an adaptive behavioral strategy, thereby facilitating functional recovery. Previous research has confirmed that exercise adherence significantly predicts lower levels of physical disability, which in turn underpins social participation [40]. Generally, within a certain range, lower level of physical disability is associated with fewer restrictions and greater frequency of social engagement.

Notably, the simple mediation pathway psychological resilience → exercise adherence → social participation satisfaction was significantly stronger than the chain mediation path psychological resilience → exercise adherence → physical disability → social participation satisfaction. This difference may be closely tied to effective emotion regulation—a process supported by neurobiological systems linked to resilience [18, 41]. Emotion regulation refers to the process through which individuals manage and modify their emotional experiences. Psychological resilience heavily relies on top-down emotional regulation mediated by the prefrontal cortex (PFC), which inhibits amygdala overactivation and prevents overwhelming negative emotions [18]. Social participation satisfaction is a more subjective and emotional dimension. Through consistent exercise (high adherence), highly resilient patients gain greater self-efficacy, perceived control, and accomplishment [42, 43]. These positive psychological experiences may directly enhance subjective satisfaction with social participation, somewhat independent of the level of physical functional improvement. This also suggests the possibility of a “threshold effect” in physical function gains—beyond a certain point, further functional improvement may contribute marginally to satisfaction. In contrast, the positive meaning derived from adherence to exercise itself may contribute more directly and strongly to satisfaction.

### Implications

This study confirms that psychological resilience influences social participation through exercise adherence and physical disability. Rehabilitation interventions should therefore adopt an integrated approach, focusing not only on physical recovery but also on psychological support. Moreover, fostering intrinsic motivation for exercise is far more effective than passive or externally imposed treatment. Established strategies for enhancing psychological resilience-such as Cognitive Behavioral Therapy (CBT), Acceptance and Commitment Therapy (ACT), and structured psychological resilience training-should be incorporated into routine rehabilitation [44, 45]. Interventions including goal setting, motivational interviewing, family involvement, and constructive feedback may improve exercise adherence [46], thereby supporting both functional recovery and life satisfaction.

### Limitations

This study has several limitations. First, although the cross-sectional design is a pragmatic approach for initial testing of mediation hypotheses, it precludes causal inferences due to the lack of temporal precedence, which is essential for establishing mediation. While reciprocal relationships among resilience, adherence, function, and participation are theoretically plausible [47, 48], we did not perform reverse mediation analyses because cross-sectional data cannot establish temporal precedence [49]. Future longitudinal or intervention studies are needed to validate the causal pathways and examine potential bidirectional dynamics. Second, participants were recruited from rehabilitation units of a single hospital in Shanghai, which may limit the generalizability of the findings. Finally, only exercise adherence and physical disability were examined as mediators; other potential factors such as social support, depression, or anxiety should be explored in future research.

## Conclusion

This study clarifies the mechanisms by which psychological resilience promotes social participation in stroke survivors with disability, highlighting the chain-mediated effects of exercise adherence and physical disability. Exercise adherence was identified as a key mediator between psychological resilience and participation satisfaction. These results support the implementation of resilience-based interventions aimed at promoting exercise adherence, thereby fostering social participation in this population.

## Data Availability

Data supporting this research is available upon reasonable request. Contact the corresponding author for access.
